# gC1qR: A New Target for Cancer Immunotherapy

**DOI:** 10.3389/fimmu.2023.1095943

**Published:** 2023-01-26

**Authors:** Yanna Lei, Xiaoyu Li, Diyuan Qin, Yugu Zhang, Yongsheng Wang

**Affiliations:** ^1^ Thoracic Oncology Ward, Cancer Center, West China Hospital, Sichuan University, Chengdu, Sichuan, China; ^2^ State Key Laboratory of Biotherapy, Sichuan University, Chengdu, Sichuan, China; ^3^ Clinical Trial Center, National Medical Products Administration Key Laboratory for Clinical Research and Evaluation of Innovative Drugs, West China Hospital, Sichuan University, Chengdu, Sichuan, China

**Keywords:** gC1qR, cancer, immunotherapy, tumor microenvironment, antiangiogenic

## Abstract

Although breakthroughs in cancer treatment have been achieved, immunotherapy yields only modest benefits in most patients. There is still a gap in clarifying the immune evasiveness and immune-resistance mechanisms. Identifying other candidate targets for cancer immunotherapy is therefore a clear unmet clinical need. The complement system, a pillar of innate immunity, has recently entered the limelight due to its immunoregulatory functions in the tumor microenvironment (TME). In particular, gC1qR, a receptor for globular heads of C1q, serves as a promising new target and has attracted more attention. gC1qR, also named P32/C1qBP/HABP1, is a multifunctional protein that is overexpressed in various cancers and holds prognostic value. It regulates the tumorigenic, progression and metastatic properties of tumor cells through several downstream signaling pathways, including the Wnt/β-catenin, PKC–NF-κB and Akt/PKB pathways. A few preclinical experiments conducted through gC1qR interventions, such as monoclonal antibody, chimeric antigen receptor T‐cell (CAR‐T) therapy, and tumor vaccination, have shown encouraging results in anticancer activity. The efficacy may rely on the regulatory role on the TME, induction of tumor cells apoptosis and antiangiogenic activity. Nevertheless, the current understanding of the relationship between cancer immunotherapy and gC1qR remains elusive and often contradictory, posing both opportunities and challenges for therapeutic translation in the clinic. In this review, we focus on the current understanding of gC1qR function in cancer immunology and highlight the vital roles in regulating the TME. We also examines the rationale behind targeting gC1qR and discusses the potential for translating into clinical practice.

## Introduction

Immune checkpoint inhibitors (ICIs) therapy targeting cytotoxic T lymphocyte antigen 4 (CTLA4) or programmed cell death-1 (PD-1) and its ligand PD-L1 has become the backbone of treatment for many cancers (1–3). Immune checkpoints are mainly expressed on the immune cells and tumor cells and inhibit the activation of immune system and help tumors escape from the host immune attack (4, 5). ICIs, therefore, could block the negative signaling on T-cell activation from immune checkpoints and potentiate antitumor T-cell activity (6, 7). However, the majority of patients exhibit poor or limited responses to these therapies even in combination, do not derive clinical benefit regardless of the cancer type, which highlights the necessity for finding alternative targets to gain optimal patient benefits (8, 9). The complement system is an integral part of innate immunity that consists of a network of plasma and membrane proteins (10). Complement could protect against nonself material, such as aberrant endogenous proteins and bacteria, by orchestrating the immune response through opsonization, recruitment of immune cells to the site of infection and causing cell lysis (11, 12). Recent studies have revealed the immunoregulatory functions of complement in cancer immunotherapy (13, 14). Complement regulates signaling pathway of tumor cells, thus promoting tumor growth, invasion and metastasis. Moreover, the complement system also correlates with angiogenesis, stromal composition and immune responses (15).

gC1qR, also named P32/C1qBP/HABP1, a receptor for globular heads of C1q, has begun to garner significant interest in the immune-oncology field as a novel potential target (16, 17). This protein bound with high affinity to the globular heads of C1q under physiological ionic strength (18). Under normal physiology, the gC1qR engages in a wide range of physiological activities, including mitochondrial metabolism and dynamics, apoptosis, splicing, immunological response, and inflammation (18). It binds to a plethora of proteins found in plasma, on the cell surface and on pathogenic microorganisms. In contrast, in cancer conditions, gC1qR played a vital role in cancer progression and correlated with patient prognosis (19). gC1qR could reshape the tumor microenvironment (TME) by modulating immune cells and cancer cells, resulting in poor anti-tumor efficacy. The activity of targeting gC1qR has been explored in CAR‐T therapy, monoclonal antibodies and cancer which showed effective anti-tumour immune responses (20, 21). Here, we focus on recent advances in the understanding of gC1qR in cancer, review results supporting the role of gC1qR in cancer immunology and illustrate its potential for translation into clinical practice.

## Structure, expression, and function of gC1qR

gC1qR has several names because it was discovered by three distinct groups independently. HABP1 was first identified as a glycoprotein containing sialic acid by D’Souza and Datta in 1985 (22). Subsequently, studies have established that the HABP1 was involved in many regulatory processes related to hyaluronan (HA), such as adhesive function and regulatory role in reproduction (23). In 1991, Krainer and collaborators described that P32, which co-purified with the splicing factor SF2, had the same cDNA sequence as HABP1 (24). gC1q-R is a highly conserved, acidic protein (pI=4.15), which was first isolated from membranes of the lymphoblastoid cell line Raji and identified as a receptor for globular heads of C1q (25, 26). The amino acid sequences of HABP1, P32, and gC1qR were identical, and they were all codified by the same gene. The gC1qR gene is located on chromosome 17p13.3 in humans, and chromosome 11 in mice. The gene is highly conserved and the cDNA sequence between the human and rodent genes is almost identical (~89.9%) (27).

The molecular weight of gC1qR in SDS-PAGE is about 33 kDa. Under non-denaturing and non-dissociating conditions, it is a doughnut-shaped trimer of 3 identical chains with 97.2kDa (18). The formation of multimers may be crucial to enhancing its affinity for multivalent ligands, such as C1q and high molecular weight kininogen (HK) (28). The doughnut-shaped quaternary structure has two sides. The solution side contains a high distribution of negatively charged residues, whereas the membrane face has a more or less neutral net charge (29). The protein is synthesized as a prepro-protein of 282 amino acid residues and then becomes a mature protein of 209 residues through a site-specific cleavage and removal during post-translational processing (30). It has one Cys at residue 186 and thus does not have any intrachain disulfide bonding (29). Research on the translated amino acid sequence reveals there is no conventional consensus motif or glycosylphosphatidylinositols (GPIs) anchor present (31). Hence, it may transfer signals through the association with partner transmembrane proteins (18). As research has progressed, gC1qR has been identified as a multicompartmental and multifunctional protein (32). gC1qR not only presents in the mitochondrial matrix but also localizes at all compartments of the cell, including the extracellular cell surface and nucleus (33).

The ubiquitous distribution of gC1qR suggests that it may be involved in a wide range of biological responses. In addition to combining with C1q, gC1qR could bind multiple ligands including thrombin, vitronectin, HK and factor XII (HF) (34–36). The interplay between gC1qR and its binding partners leads to the classical complement pathway activation, cell adhesion, and activation of the kinin system (37, 38). Furthermore, it regulates the homeostatic and thrombotic events (39). The contact system proteins HF, prekallikrein (PK), and HK comprise the initiators of the so-called intrinsic blood coagulation system (40). gC1qR could interact with HK and HF, and then activate intrinsic coagulation and kinin pathways (39, 41, 42). gC1qR also plays a critical role in the maintenance of phosphorylation (43, 44). The disruption of the p30 gene (homolog of p32 in yeast) impaired mitochondrial ATP synthesis, which could be restored by introducing human p32 cDNA. gC1qR is necessary for functional mitoribosome formation to synthesize proteins within mitochondria and induce mitochondria-dependent cell death (45, 46). In addition, gC1qR was correlated with cell apoptosis. The overexpression of gC1qR in fibroblast cells induces inhibited cell growth, extensive vacuolation, restricted entry to the S-phase, and finally leading to apoptosis (47).

Moreover, a growing number of studies have shown the link between gC1qR and virus, such as respiratory syncytial virus (RSV), hepatitis C virus (HCV) and human immunodeficiency virus (HIV-1). The gC1qR protein was present as a key factor for production of RSV and the mitochondrial localized gC1qR contributes to RSV infection (48). gC1qR has been identified as an HCV core-binding protein. The combination of gC1qR on T cells and HCV core protein suppressed the T-cell proliferation, thus further impacting the human cell–mediated immune response (49). And the inhibition of gC1qR could reverse the T-cell responsiveness. The gC1qR-mediated immune suppression in HCV also confirmed in other studies, the interaction between gC1qR and HCV core protein also influenced the Th1 differentiation of CD4+ T cells by inhibiting the dendritic cell IL-12 production (50). In addition, the gC1qR also participated in the HIV-1 pathogenesis (51). These results highlight the critical role gC1qR plays in a growing list of diseases.

## gC1qR in cancer

### The role of gC1qR in cancer diagnosis, prognosis, and putative signaling pathways

**Figure 1:**
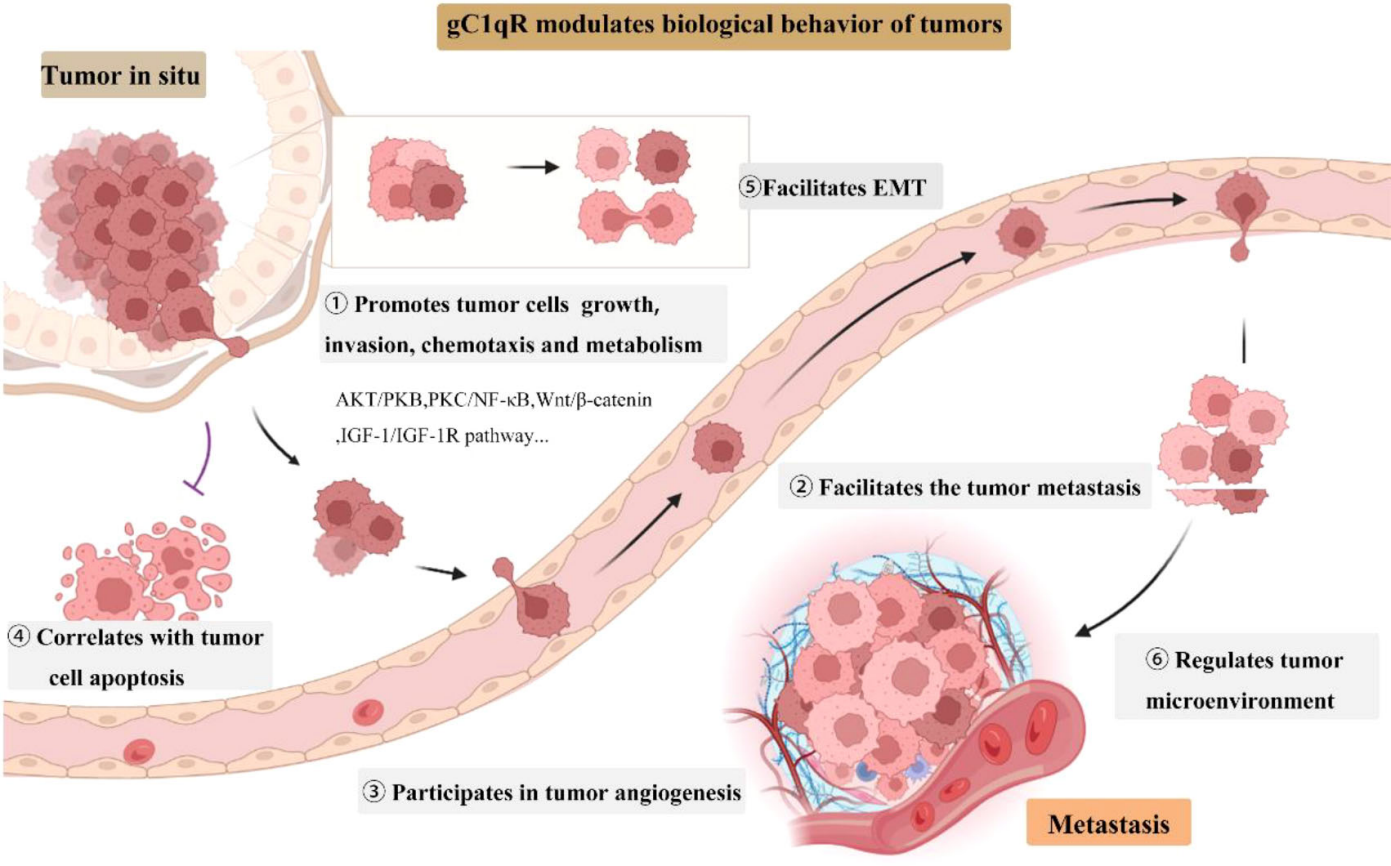
gC1qR roles in tumor growth, invasion, and progression. EMT, epithelial to mesenchymal transition.

Recent researches have shown that gC1qR contributes to cancer progression, invasion and metastasis, and correlates with clinicopathological features of tumors (**Figure 1**) **(** 23, 52–55). Its overexpression was found in multiple cancer cells, including breast, ovarian, prostate, melanoma, lung, pancreatic, colon cancer, and malignant pleural mesothelioma (55–58). It has been reported the expression level of gC1qR was correlated with tumor stage, grade, tumor size, and clinical outcome (59, 60). In breast cancer, gC1qR overexpression was significantly related to distant metastasis, higher TNM stages, increased tumor size, axillary node metastasis and poor survival (23, 60). A poor outcome was also found in patients with ovarian cancer with gC1qR overexpression (61). Similar observations were reported in patients with gastric cancer (62) and endometrial cancer (63). Additionally, it is involved in cancer cell chemotaxis and metabolism. gC1qR modulates the cancer cell chemotaxis through binding to protein kinase C ζ (64, 65). Active caspase-1 cleaves gC1qR and then promotes aerobic glycolysis in tumor cells and boosts carcinogenesis (66). However, its roles may vary according to different cancer types. In renal cell carcinoma (RCC), higher expression levels of Y-box-binding protein 1 (YBX1) and lower expression of gC1qR were found in tumor tissues (67). And the gC1qR negatively regulates the activation of YBX1 which is closely associated with tumorigenesis.

In addition to tumor cellular gC1qR expression, soluble gC1qR (sgC1qR) was also discovered. The researchers observed an increased level of soluble gC1qR in metastatic pancreatic cancer patients were noted with disease progression (68). Soluble gC1qR was also detected in malignant pleural and peritoneal effusions and the level was higher in malignant effusions than serum. Another finding demonstrated that soluble gC1qR may serve as an autocrine growth signal for cancer cell proliferation (69).

gC1qR is involved in multiple signaling pathways to modulate the biological behavior of tumors. In HepG2 cells, gC1qR overexpression boosts the cell proliferation through up-regulation of cyclin D1 in AKT-dependent pathway (70). Sinha S et al. revealed the gC1qR regulates cell proliferation, migration, and invasion in melanoma by regulating AKT/PKB signaling and altering oncogenes as well as epithelial to mesenchymal transition (EMT) markers in both mouse and human melanoma (71). In addition, gC1qR also induced the NF-kappa B dependent MMP-2 activation through integrin α_v_β_3_ interaction to regulate the cell migration and tumor development (72). Depletion of gC1qR in triple-negative breast cancer (TNBC) inhibits hypoxia-induced activation of the PKC/NF-KB/VCAM-1 signaling pathway, resulting in cancer cell metastasis blocking (73). The Wnt/β-catenin pathway also correlated with gC1qR. Circular RNA MTCL1 could regulate the Wnt/β-catenin pathway through interacting with gC1qR and cause the laryngeal squamous cell carcinoma progression (74). Moreover, gC1qR mediates hepatic metastasis of pancreatic cancer *via* IGF-1/IGF-1R signaling (57). However, another contradictory study indicated that gC1qR may serve as a tumor suppresser by modulating the p-GSK3/β-Catenin/L1CAM expression in RCC (75).

To summarize, gC1qR plays a pivotal role in the growth, survival, and metastasis of tumor cells. It also serves as a novel marker for cancer prognosis and diagnosis, provides new opportunities for cancer therapy although the regulatory mechanisms of gC1qR in various cancers have not yet been elucidated.

### gC1qR and the tumor microenvironment

TME, which consists of immune cells, non-tumorigenic stromal cells, and tumor cells, is the key to carcinogenesis and associates with immune resistance as well as immune evasion (76). Despite some conflicting conclusions, accumulating evidence suggests that gC1qR may serve as a potential regulator of TME interactions.

gC1qR has been proved to be essential for CD8^+^ T cell survival, proliferation, and anti-tumor immune function (20, 77). It regulates the epigenetic program and promotes the dynamic transcriptional program of effector CD8^+^ T cells. gC1qR increases T cell proliferation through regulating the AKT‐mTORC1 signaling pathway and improving T cell survival by recruiting anti-apoptotic proteins such as Bcl2 and BclXL, thereby inhibiting caspase3 cleavage and PARP inactivation (20). gC1qR deficiency impedes T cell proliferation, hinders the CD4^+^ and CD8^+^ T cell infiltration and aggravates tumor infiltrating T cell exhaustion (20). Additionally, gC1qR knockdown aggravated the exhausted phenotype of CD4^+^ and CD8^+^ T cells through increasing co–inhibitory molecules such as PD‐1, Tim‐3, and LAG‐3. gC1qR knockdown also impaired the efficacy of CAR-T cells (20). gC1qR has been shown to be required for the dendritic cells (DCs) metabolism and maturation (78). But the relationship between DCs and gC1qR in cancer immunotherapy has not been elucidated. Fogal et al. also demonstrated that the cell-surface gC1qR could serve as the marker for tumor-associated macrophages/myeloid cells. They found gC1qR is the receptor for LyP-1 and the p32/LyP-1 positive cells were also positive for macrophage/myeloid cell markers (79). gC1qR also acts on the macrophages and leukocytes infiltration in TME and gC1qR knockdown inhibits the infiltration of these cells (71).

Angiogenesis, a key component of the TME, is necessary for tumor invasion and metastasis. Abnormal tumor blood vessels lead to hypoxia and contribute to inhibitory TME (80). gC1qR was identified to be expressed on the cell surface of activated (angiogenic) endothelial cells and evidence showed that the gC1qR may participate in tumor angiogenesis, thereby modulating the TME (71, 81, 82). The neutralization of cell-surface gC1qR with antibody inhibits vascular endothelial growth factor (VEGF) signaling and prevents angiogenesis in human umbilical vein endothelial cells (HUVECs) (81). Another study also revealed gC1qR-silenced tumors showed decreased proliferation and angiogenesis compared to control tumors in melanoma (71). Those results suggested the gC1qR may function as an angiogenic-related protein and serve as a potential therapeutic target for blocking tumor angiogenesis. Hypoxia is a widely established factor of the TME that could upregulate the hypoxia-inducible factor-1α (HIF-1α) and PD-L1 expression, and promote immune escape and immune resistance (83). Studies showed that the expression of gC1qR was enhanced by HIF-1α (73) and increased expression of gC1qR was found in hypoxia area of tumor (79). gC1qR, also a novel receptor of Hyaluronic acid (HA), could interact with HA and regulated HA-mediated cellular event as well as affect behavior of tumor cells. The extracellular matrix (ECM) is a dynamic network that serves as a highly active part of the TME (84). HA is one of major ECM components which relates to the cell–cell contacts such as adhesion, motility, and differentiation and cancer metastasis (85).

These results serve to illustrate the conflicting role of gC1qR on TME. gC1qR knockdown may modulate T cells’ mitochondrial fitness to decrease the infiltration and anti-tumor function, but the blockage of cell-surface gC1qR could decrease the blood supply of tumor. Although the exact role of gC1qR in TME has not been fully validated, it shows potential to regulate tumor cells, immune cells, angiogenesis and other components of the TME.

## Potential for translating into clinic

### Advances in preclinical research

**Figure 2:**
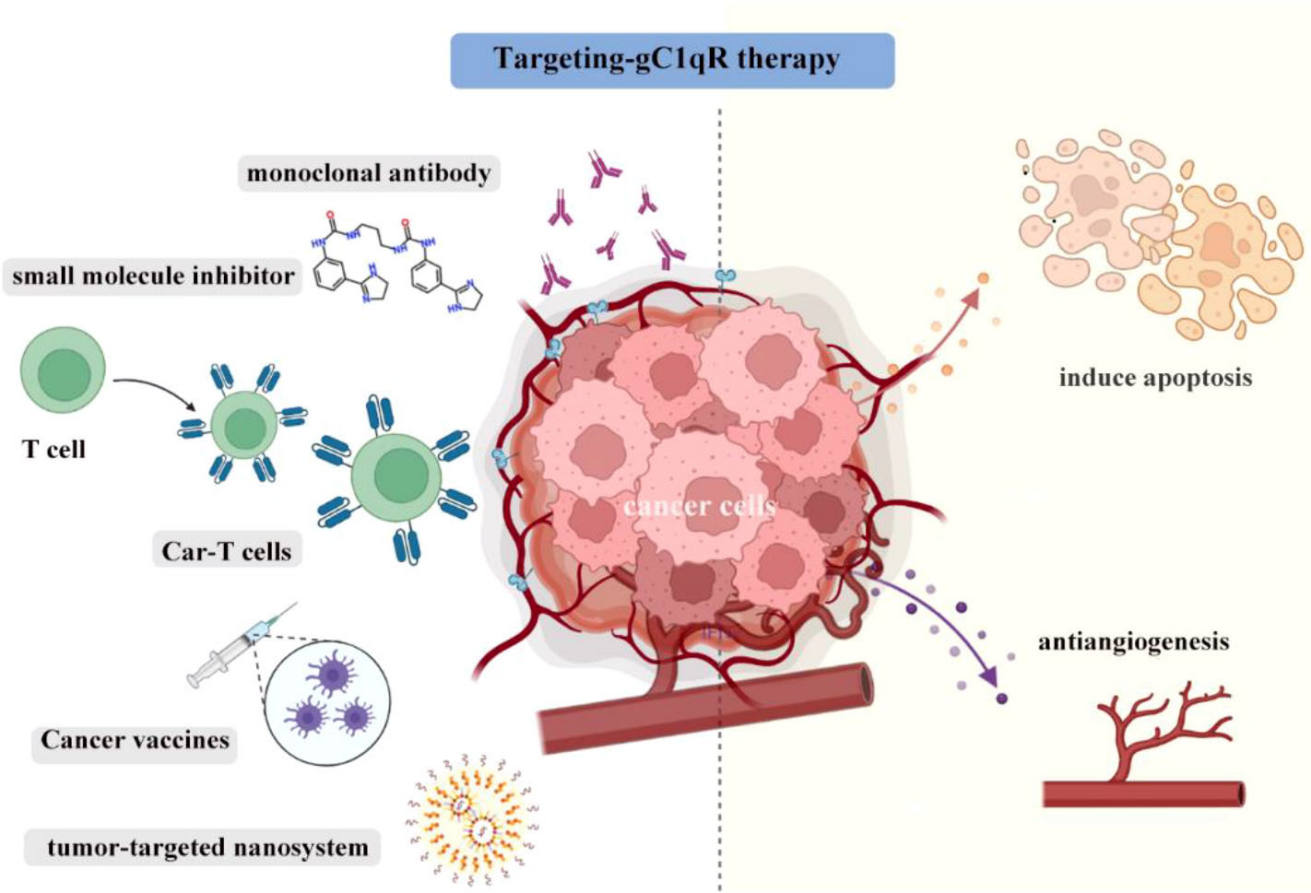
The gC1qR blockade can be largely classified into monoclonal antibody therapy, highly selective small molecules, CAR-T therapy, tumor vaccination and used in nanosystem to kill tumors. Induction of tumor cells apoptosis and antiangiogenic activity are key element in successful and efficient gC1qR targeting.

The complex function of gC1qR in tumor regulation and immunity poses opportunities for therapeutic translation in cancer immunotherapy. Studies on preclinical mouse tumor models have indicated that gC1qR can be considered as a suitable target for different cancer therapy approaches, such as monoclonal antibody therapy, small molecules, CAR-T technology, and tumor vaccination (**Figure 2**).

In TNBC, gC1qR monoclonal antibody 60.11-treated mice have smaller tumors than control mice. Mechanistically, mice in treatment group had higher expression levels of apoptosis-related markers and decreased CD31 (angiogenesis-related marker) expression (21). In other words, the antibody targeting gC1qR promoted tumor cell apoptosis and decreased angiogenesis. Potential toxicity was also evaluated and the results showed that there was no evidence of tissue damage observed on vital organs. As gC1qR was overexpressed in malignant pleural mesothelioma and mesothelioma (MSTO) cells lines, thus the antitumor efficacy gC1qR monoclonal antibody 60.11 was also assessed in MSTO mice models. The results indicated that gC1qR monoclonal antibody 60.11 reduced mesothelioma tumor volume *via* increasing apoptosis and reducing neovascularization (19). Additionally, the gC1qR-neutralizing antibody 3D9 could prevent the cells migration of plenty of cancer cell lines, including A549, MCF7 and MDA-MB-231. It also inhibits the growth factor-stimulated lamellipodia formation and receptor tyrosine kinases (RTKs) signaling (81). More importantly, 3D9 also had an anti-angiogenic effect and prevented lamellipodia formation, cell migration and VEGF signaling in HUVECs. *In vivo*, 3D9 significantly inhibited the tumor growth in A549 tumor-bearing nude mice (81). Above results confirmed the anti-tumor efficacy of gC1qR monoclonal antibody in cancer immunotherapy, but it is worth noting that in some specific tumors such as the glioblastoma, the blood–brain barrier may decrease the antitumor activity. To solve this problem and to specifically target the mitochondrial-localized gC1qR, Yenugonda et al. identified a highly selective and brain-penetrant small molecule M36 (86). M36 was selective for gC1qR overexpressing cells. It effectively impaired the growth of glioma cells, and sensitized the tumor cells to glucose depletion.

CAR-T technology has developed rapidly in recent years and is one of the most promising strategies in cancer therapy (87). Rousso-Noori et al. designed gC1qR CAR T cells in glioma after confirming that gC1qR was expressed in mice and human glioma. The novel gC1qR CAR-T therapy significantly induces tumor regression in both syngeneic and xenograft models, and extended the overall survival of mice (88). Consistent with the above findings, the gC1qR CAR T cells also had antiangiogenic activity in this study.

Cancer vaccines hold promise as an immunotherapeutic modality and it could induce long-term immunological memory for antitumor responses (89, 90). Dehghan-Manshadi et al. designed vaccine based on peptides derived from mouse gC1qR protein and the results showed that compared with the control group, mice in the vaccinated group had higher levels of IFN-γ and perforin, lower tumor size and longer survival time (91). Additionally, they also found that forkhead box P3 (Foxp3) gene was significantly down-regulated and Fas ligand (FasL) gene expression was up-regulated in splenocytes vaccinated mice group (91).

Nanotechnology has recently attracted significant attention worldwide for cancer treatment (92). Previous research revealed the gC1qR was the receptor for tumor-homing peptide LyP-1 which could specifically bind to tumor lymphatics and tumor cells (79, 93, 94). LyP-1 has been used to deliver nanoparticles to breast cancer with gC1qR overexpression and effectively reduced tumor growth *in vivo (* 86, 95). Sharma S et al. also described a tumor-penetrating nanosystem that strongly inhibits breast tumor growth in mice, in which a homing component targets tumor vessels through binding gC1qR (82). In this context, targeting gC1qR seems to be an increasingly promising strategy in the field of cancer therapy.

### Questions and challenges

Considerable insight into its function and therapeutic potential have been gained until now. However, key questions remain regarding fundamental gC1qR biology and translational application. First, anti-gC1qR monotherapy has shown anti-tumor activity in pre-clinical murine models, but such activity in humans is still unknown. As animal models cannot mimic the complex environment of human body, thus, well designed clinical trials are essential to demonstrate the efficacy and safety of anti-gC1qR in human beings. Moreover, monotherapy often displays important drawbacks and given the promise of ICI-based combination therapy (96, 97), the efficacy of ICIs and anti-gC1qR therapy also demand investigation. Second, the exact role of gC1qR in the regulation of the TME is still not sufficiently elucidated and current knowledge on this target is not enough. Determination of gC1qR expression in human T-cell subsets especially tumor-infiltrating lymphocytes and comprehensive validation of related functional mechanisms in immune cells are prerequisites for the development of anti-gC1qR therapeutics. Additionally, gC1qR is also expressed on the normal cells (98), the safety of targeting gC1qR should be assessed carefully in human body, although no obvious side effects were revealed in mice. Finally, targeting gC1qR has just started as a new field within cancer immunotherapy and further *in vitro* and *in vivo* studies are still needed. Based on abundant and solid evidence, clinical trials are the next step directions and targeting gC1qR might benefit selected patient populations.

## Conclusion

Despite ICIs having led to unprecedented breakthroughs in cancer treatment, most patients do not respond well to ICIs. Such results pave the way for targeting other promising immune checkpoints. In this review, we provide a strong foundation for targeting the gC1qR to be a novel anticancer therapeutic approach and hold promise for translate into the clinic, although there still have no related clinical trial to date. Accumulating evidence has shown that gC1qR modulates tumor growth, invasion, and progression. It participates in multiple signaling pathways and regulates the TME. The gC1qR blockade can be largely classified into monoclonal antibody therapy, highly selective small molecules, CAR-T therapy, tumor vaccination and nanoparticle strategies for cancer therapy. Considering the characteristics as presented here, it seems that the regulation role on TME, induces the apoptosis of tumor cells and antiangiogenic activity are key elements in successful and efficient gC1qR targeting. However, the complex function of gC1qR poses both opportunities and challenges for therapeutic translation in clinic. More efforts should be devoted to accelerating the development of gC1qR inhibitors for clinical use.

## Author contributions

YW: Conceptualization. YL, XL: Original draft preparation. all authors contributed to the article and approved the submitted version.
